# Ethoflow: Computer Vision and Artificial Intelligence-Based Software for Automatic Behavior Analysis

**DOI:** 10.3390/s21093237

**Published:** 2021-05-07

**Authors:** Rodrigo Cupertino Bernardes, Maria Augusta Pereira Lima, Raul Narciso Carvalho Guedes, Clíssia Barboza da Silva, Gustavo Ferreira Martins

**Affiliations:** 1 Department of Entomology, Federal University of Viçosa, Viçosa 36570-900, MG, Brazil; guedes@ufv.br; 2 Department of Animal Biology, Federal University of Viçosa, Viçosa 36570-900, MG, Brazil; maugusta@ufv.br; 3 Laboratory of Radiobiology and Environment, University of São Paulo-Center for Nuclear Energy in Agriculture, 303 Centenário Avenue, Piracicaba 13416-000, SP, Brazil; clissia@usp.br; 4 Department of General Biology, Federal University of Viçosa, Viçosa 36570-900, MG, Brazil; gmartins@ufv.br

**Keywords:** animal monitoring, convolutional neural networks, deep learning, machine learning, object detection, tracking

## Abstract

Manual monitoring of animal behavior is time-consuming and prone to bias. An alternative to such limitations is using computational resources in behavioral assessments, such as tracking systems, to facilitate accurate and long-term evaluations. There is a demand for robust software that addresses analysis in heterogeneous environments (such as in field conditions) and evaluates multiple individuals in groups while maintaining their identities. The Ethoflow software was developed using computer vision and artificial intelligence (AI) tools to monitor various behavioral parameters automatically. An object detection algorithm based on instance segmentation was implemented, allowing behavior monitoring in the field under heterogeneous environments. Moreover, a convolutional neural network was implemented to assess complex behaviors expanding behavior analyses’ possibilities. The heuristics used to generate training data for the AI models automatically are described, and the models trained with these datasets exhibited high accuracy in detecting individuals in heterogeneous environments and assessing complex behavior. Ethoflow was employed for kinematic assessments and to detect trophallaxis in social bees. The software was developed in desktop applications and had a graphical user interface. In the Ethoflow algorithm, the processing with AI is separate from the other modules, facilitating measurements on an ordinary computer and complex behavior assessing on machines with graphics processing units. Ethoflow is a useful support tool for applications in biology and related fields.

## 1. Introduction

Behavioral studies are critical to understanding the fundamental aspects of animal ecology [1,2]. The assessment of animal behavior by visual inspection is limited and subjective and does not allow observations over long periods [3]. The use of computational tools in behavioral assessments allows accurate and long-term evaluations of animals [2,4]. For instance, automatic tracking systems obtain the animal’s position in each frame of a digital video and record the Cartesian or polar coordinates of the movement [5].

From animals’ coordinates over time is possible to calculate important kinematic measurements (e.g., the animal walked distance and meandering). Furthermore, evaluating complex behaviors (measurements based on characteristics extracted from specific animal behaviors) can provide relevant insights into animal biology. For example, the evaluation of complex behaviors among social insects, such as changes in trophallaxis (the complex social behavior of food exchange among nestmates), is important for understanding their response to stress agents such as pesticides [6,7].

Robust systems are needed for animal monitoring in heterogeneous environments (i.e., complex environmental landscapes such as in the field or multi-scenes with variation in color, luminosity, texture, and different objects) [2]. The greatest challenge in heterogeneous environments involves extracting target objects from the background (segmentation) [8]. Background subtraction or thresholding are well-established in digital image processing for object segmentation [5]. However, these approaches require video recordings of homogeneous environments (i.e., with similar pixel values or slight variation in color, luminosity, and texture) and are not applicable in heterogeneous environments.

Using artificial intelligence (AI) technics such as machine and deep learning can be sufficiently robust for animal behavior assessments in heterogeneous environments [9]. Convolutional neural networks (CNNs) are deep learning models widely used in computer vision [10]. These models are organized into layers composed of several neurons and convolutional kernels/filters with learnable weights. The CNNs comprise two basic parts: a convolutional base and a densely connected classifier. In the convolutional base, operations (convolutions) decompose the input in abstract and useful information (feature extraction) for classification in dense layers. Thus, the convolutional base’s function is finding appropriate representations (feature map) for the classification in the dense layers, where the feature map undergoes successive nonlinear operations to obtain the predictions. The learning process of neural networks consists of updating the network parameters in the opposite direction of the cost function gradient, reducing the loss, until finding optimal parameters that result in a minimal loss (i.e., minimal difference between the expected value and the predicted value) [10].

Given the potential application of AI and the demand for studying animal behavior in natural conditions [2], we developed the open-source desktop software Ethoflow. In the software algorithm, (i) we used unsupervised machine learning to provides an optimal identity assignment and maintain the identity among individuals in animal group tracking. Using deep learning, (ii) we implemented instance segmentation for animal monitoring in heterogeneous environments. Moreover, (iii) deep learning was applied to recognize animal complex behaviors. Besides, (iv) we performed bioassays with two species of eusocial bees to validate Ethoflow. Finally, (v) we evaluated parameters associated with Ethoflow’s performance. Thus, the proposed software:

- has a graphical user interface (GUI) and has already been successfully applied in other studies [11,12];
- performs animal tracking in homogeneous or heterogeneous environments;
- can maintain the identity among individuals in animal group tracking;
- evaluates various kinematic variables (e.g., mean speed, turning angle, and group interaction);
- supports complex behavior assessment (e.g., mating, grooming, and trophallaxis).

A brief overview of recent tools involving tracking methods and AI techniques for animal behavioral assessments is presented in Section 2. The methods and results of the Ethoflow algorithm, applications in different setups and bioassays, and performance in processing speed and accuracy are described in Section 3 and Section 4. Finally, the discussion and conclusions are presented in Section 5 and Section 6, respectively.

## 2. Related Work

The tracking software Tracktor uses unsupervised machine learning to track animal groups maintaining individuals’ identities [13]. This software exhibited advantages in processing speed and robustness compared to the software IdTracker [14] and the ToxTrac [15]. Some other tracking software exhibit outstanding performance using deep learning algorithms [16], including the idtracker.ai [17] and the TRex [18]. These two software also apply CNNs to track many animals simultaneously with high accuracy in maintaining individuals’ identities.

In addition to tracking software, there are also tools for measuring the geometrical configuration of body parts denoted as pose estimation [16]. Deep learning approaches have also led to notable improvements in pose estimation software (e.g., DeepPoseKit, DeepLabCut, and LEAP) [19,20,21]. For instance, the DeepLabCut uses transfer learning with a pre-trained network in large datasets (e.g., ImageNet). This approach can improve performance and reduce the number of required training examples [19]. However, it may come with the cost of slow inference due to excessive parameterization in large networks. The LEAP framework uses a relatively simple 15 layers CNN to limit model complexity and maximize inference speed [20]. However, the LEAP achieved limited accuracy compared to the DeepPoseKit and DeepLabCut [21]. To improve the speed-accuracy tradeoff in DeepLabCut and LEAP, the DeepPoseKit toolkit was developed using Stacked DenseNet, a deep learning architecture that provides fast and accurate detection even at low spatial resolutions [21].

The unfolding of behavioral assessments in tools without graphical user interface (GUI) (e.g., Tracktor) [13] requires familiarity with programming, which can limit the general public use. In this context, the Ethoflow software looks user-friendly due to the GUI. The available tracking software measure large collectively animal groups with high accuracy, especially those using deep learning. However, these tracking software operate by background subtraction or thresholding [13,14,15,17,18]. These approaches require video recordings of homogeneous environments and are not applicable in the field. In Ethoflow, we implemented thresholding by Otsu’s method [22] (Section 3.1.3) to handle assessments in a homogeneous environment. In addition, we also implemented instance segmentation by Mask R-CNN [9] for evaluation in heterogeneous environments (Section 3.1.4).

With pose estimation toolkits, variables can be measured to predict complex animal behavior after some posterior machine learning analysis [23]. Although our goal with the Ethoflow software is tracking analysis, Ethoflow also directly measures complex behaviors. After hyperparameter optimization, we defined a parsimonious CNN architecture to assess complex binary behavior (Section 3.1.8). Deep learning software is computationally costly and requires graphics processing unit (GPU) hardware. Accordingly, they are not feasible to use on an ordinary computer. An interesting feature in our proposal is that the deep learning algorithms (used for analysis in a heterogeneous environment and measurement of complex behaviors) are separate from the other modules in Ethoflow. Wherefore, Ethoflow covers kinematic measurements on an ordinary computer and assesses more complex behavior with a GPU.

## 3. Materials and Methods

### 3.1. Software Features and Algorithm

**Figure 1:**
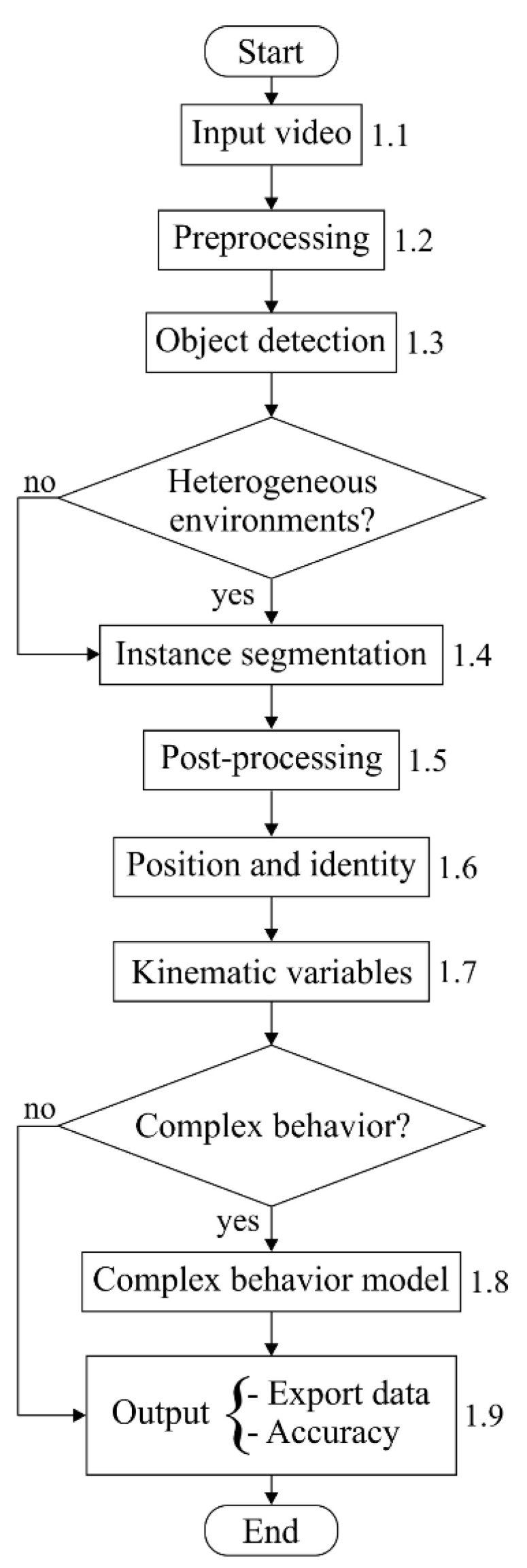
Flowchart of the Ethoflow algorithm. The numbers on the right side of the rectangles indicate the steps in the algorithm process. These steps are described in the subsequent sections from Section 3.1.1, Section 3.1.2, Section 3.1.3, Section 3.1.4, Section 3.1.5, Section 3.1.6, Section 3.1.7, Section 3.1.8 and Section 3.1.9. Diamond symbols indicate the option of using the deep learning algorithms (for analysis in a heterogeneous environment or measurement of complex behaviors) according to the need. Thus, the Ethoflow performs kinematic measurements on an ordinary computer and assesses more complex behavior with a graphics processing unit hardware.

The Ethoflow software was developed in modality desktop application with Python language, including the image library OpenCV [24] and the framework TensorFlow [25] with Keras for AI models. Other libraries, such as SciPy [26], Numpy [27], Pandas [28], and SciKit Learn [29], were also used. We recommended Python version 3.6.8 and Microsoft Windows 10 when running the Ethoflow. The main input and output files, formats, descriptions, and quick examples of using these Ethoflow files are described in Supplementary materials (Table S1). The following subsections will provide further details on the Ethoflow algorithm steps (Figure 1).

#### 3.1.1. Input Video

Multi-threaded processing was implemented in the algorithm. In this procedure, the video is read in a thread independent of the processing thread, and the frames are stored in a stack (Figure 1; step 1.1; Appendix A). This avoids the delay between frame reading and other processing steps of the algorithm, whereby frames are always available to obtain better rates in frames per second (fps).

#### 3.1.2. Preprocessing

In preprocessing (Figure 1; step 1.2), the video is processed to eliminate the regions that are not of interest to the user and transformed into a virtual primary color system (color space XYZ). In this color space, the chromaticity (XZ) and luminance (Y) are coded separately, resulting in a more uniform response to the luminosity variation. Then, grayscale transformation and normalization are applied to increase homogeneity between the frames. Smoothing is also applied through a transformation based on the median of neighborhood pixels to eliminate noise.

#### 3.1.3. Object Detection

After preprocessing, manual and automatic image thresholds are applied to detect individuals (Figure 1; step 1.3). In manual thresholding, the classification of pixel (x,y) is performed according to a global threshold defined by the user (*g*):

(1) $$f\left(x,y\right)=\left\{\begin{array}{c} 1\;if\left(x,y\right)>g\\ 0\;if\left(x,y\right)\leq g \end{array}\right\}.$$

One of the automatic thresholding options is based on Otsu’s method [22], wherein the optimal threshold minimizes the within-class variance. This algorithm attempts to find a threshold value (*k*) that minimizes the within-class variances $c_{0}\;\mathrm{and}\;c_{1}$ (background and objects, respectively). If the set of gray levels of an image L={1, 2,⋯,l} and the total number of pixels $N=\left\{n_{1},n_{2},\cdots ,n_{l}\right\},$ then the probability of occurrence of a gray level $\left(p_{i}\right)$ is given by

(2) $$p_{i}=\frac{n_{l}}{N}.$$

As the method is based on the normalized histogram,

(3) $$\overset{L}{\underset{i=1}{\sum }}p_{i}=1.$$

Thus, the probability of occurrence $\left(\omega_{i}\right),$ means $\left(\mu_{i}\right),$ and variances $\left(\sigma_{i}\right)$ of each class, are given by

(4) $$\omega_{0}=\overset{k}{\underset{i=1}{\sum }}p_{i}\;\mathrm{and}\;\omega_{1}=\overset{L}{\underset{i=k+1}{\sum }}p_{i},$$

(5) $$\mu_{0}=\frac{\sum_{i=1}^{k}i*p_{i}}{\omega_{0}}\;\mathrm{and}\;\mu_{1}=\frac{\sum_{i=k+1}^{L}i*p_{i}}{\omega_{1}},$$

(6) $$\sigma_{0}^{2}=\frac{\sum_{i=1}^{k}{\left(i-\mu_{0}\right)}^{2}p_{i}}{\omega_{0}}\;\mathrm{and}\;\sigma_{1}^{2}=\frac{\sum_{i=k+1}^{L}{\left(i-\mu_{1}\right)}^{2}p_{i}}{\omega_{1}}.$$

The within-class $\left(\sigma_{w}\right)$ and between-class $\left(\sigma_{b}\right)$ variances are

(7) $$\sigma_{w}^{2}=\omega_{0}\sigma_{0}^{2}+\omega_{1}\sigma_{1}^{2},$$

(8) $$\sigma_{b}^{2}=\omega_{0}\omega_{1}{\left(\mu_{1}-\mu_{0}\right)}^{2}.$$

The total variance is $\sigma_{t}^{2}=\sigma_{w}^{2}+\sigma_{b}^{2},$ and calculating the between-class variance improves the computational time because the variance between classes is based on first-order statistics (class means) [22].

#### 3.1.4. Instance Segmentation

**Figure 2:**
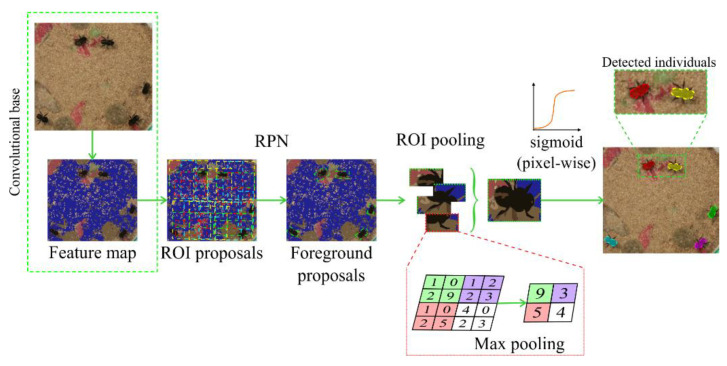
Diagram depicting the operations involved in instance segmentation used in Ethoflow for animal behavior assessments in heterogeneous environments. RPN: region proposal network; ROI: box surrounding the object of interest.

Instance segmentation (IS) [9] is another type of automatic segmentation available in Ethoflow for animal behavior assessments in heterogeneous environments (Figure 1; step 1.4). ResNet-101 [30] was the convolutional base used in this model, following a Mask R-CNN implementation [31]. In this model, the video frames pass through a convolutional base for feature extraction, leading to feature map generation. The region proposal network (RPN) is then applied, which provides several candidate boxes (ROI proposals). As several ROIs are generated, the model classifies these boxes into foreground proposals (animals) and backgrounds. ROI pooling is applied to standardize the foreground proposals’ size, slicing each foreground into a fixed number of parts, and max pooling is applied to standardize the size. Finally, the boxes labeled as real animals are instantiated using a pixel-wise sigmoid function (Figure 2).

#### 3.1.5. Post-Processing

In post-processing (Figure 1; step 1.5), morphological operations are applied to eliminate residues. First, dilation is used to fill parts that belong to the same individual but are detected separately. Second, the gradient is calculated and subtracted from the expanded frame to eliminate undesirable edges. Finally, erosion is applied to eliminate any noise erroneously detected as individuals.

#### 3.1.6. Position and Identity

In step 1.6 of the algorithm, the animal contours (the pixels contained in the animal body) are identified. The contours are identified without establishing hierarchies while retaining only the extreme points of the contour line segments. The contour measurements, such as the area, length, and the ratio between the area and length, are calculated to restrict the contours that are identified based on the user’s inputs. 

When the number of contours identified is smaller than the number of individuals specified by the user, the nonhierarchical clustering k-means algorithm is applied to separate merged individuals. In this unsupervised machine learning algorithm, the number of groups (*k*) in which the set of pixels will be grouped is equal to the number of individuals specified by the user. The initial *k* centroids are randomly defined among the set of data points. Then, the next set of centroids are chosen according to the probability of spreading between the centers [32]. The contour points are compared with each centroid and are allocated to the group where the Euclidean distance is minimal. Considering the inputs for the algorithm $X=\left\{x_{1},\ldots ,x_{n}\right\}$ of *n* data points, this algorithm runs interactively to find a set $C=\left\{c_{1},\ldots ,c_{k}\right\}$ that minimizes the function $\varphi_{x}\left(C\right)$ as follows:

(9) $$\varphi_{x}\left(C\right)=\underset{x\in X}{\sum }d{\left(x,C\right)}^{2},$$

where d(x,C)² is the distance from *x* to the closest center in *C*. To choose centroids in the k-means algorithm, the first set of centers $C_{0}$ are randomly selected from the dataset. Then, this step is repeated for 2⩽i⩽k: $c_{i}$ is chosen to be equal to a data point $x_{n}$ according to the probability [32]:

(10) $$\frac{d\left(x_{0},C\right)²}{\varphi_{x}\left(C\right)}.$$

A combinatorial optimization algorithm [33] is applied to maintain the identity of individuals, which provides the optimal identity assignment among the centroids of animal contours. This is based on the Euclidean distance between the set of centroids of the objects in the *frame_i+_*_1_ *=* $\left\{a_{1},a_{2},\ldots ,a_{n}\right\}$ and the set of centroids in the *frame_i_ =* $\left\{b_{1},b_{2},\ldots ,b_{n}\right\}$. Considering that each $a_{n}$ is assigned to only one $b_{n}$, the goal is to minimize the total cost of assignments about the distance matrix (*D*) between each $a_{n}$ and $b_{n}$:

(11) $$D=\left[\begin{array}{c} d_{1,1}d_{1,2}\cdots d_{1,n}\\ d_{2,1}d_{2,2}\cdots d_{2,n}\\ \vdots \;\vdots \;\vdots \\ d_{n,1}d_{n,2}\cdots d_{n,n} \end{array}\right].$$

The mathematical model [33] for the assignments is given as $Minimum:\sum_{x=1}^{n}\sum_{j=1}^{n}d_{ij}$, where $d_{ij}$ is the cost (Euclidean distance) from centroid $a_{n}$ to centroid $b_{n}$. There are *n!* ways to assign $a_{n}$ to $b_{n}$ and achieve the optimal assignment, interactively, with the following steps:

1. The minimum of each row is subtracted from the entire row.
2. The minimum of each column is subtracted from the entire column.
3. All zeros in the matrix are crossed with the minimum possible lines.

*If* crossing lines = *n*, then the optimal assignment is found.

*Else*: 

To determine the smallest entry not crossed by any line, 

Subtract this entry from each uncrossed row and add it to each crossed column.

Proceed to step 3.

#### 3.1.7. Kinematic Variables

Among the identified and assigned animal contours, each individual’s centroid (Cartesian position) is determined. Based on this Cartesian position *x*, *y* of individuals over time (video frames; *f*), various kinematic variables are computed in algorithm step 1.7. The distance that an animal walks during the video is tracked distance (*td*) (Equation (12)). Dividing *td* by the total time of the video, the mean velocity can be calculated. Ethoflow also calculates the maximum velocity achieved by the animal.

(12) $$td=\overset{f}{\underset{i=1}{\sum }}\sqrt{{\left(x_{i+1}-x_{i}\right)}^{2}+{\left(y_{i+1}-y_{i}\right)}^{2}.}$$

The average angle that the individual rotated in each frame (turning angle; *ta*) is computed by the absolute sum of the angles (°) of the movement divided by the video frames (*f*) (Equation (13)), while the meandering (the average angle that the individual rotated during the video; *M*) is divided by tracked distance (*td*) (Equation (14)); the angle of the movement is the arctangent of the locomotion in planes *y* (Δ*y_i_*) and *x* (Δ*x_i_*).

(13) $$ta=\frac{1}{n}\overset{f}{\underset{i=1}{\sum }}\left|\left(\frac{arctan\left(\frac{\Delta y_{i}}{\Delta x_{i}}\right)180}{\pi }\right)\right|.$$

(14) $$M=\frac{1}{td}\overset{f}{\underset{i=1}{\sum }}\left|\left(\frac{arctan\left(\frac{\Delta y_{i}}{\Delta x_{i}}\right)180}{\pi }\right)\right|.$$

The movement of individuals is categorized based on the user-defined values. When defining the analysis protocol, the user defines the thresholds for low (*tl*) and high movement (*th*). Thus, considering the movement of individuals in each frame as *mf*: *mf* ≤ *tl* is counted as resting (the time associated with no activity of the individual); *tl* < *mf* ≤ *th* is counted as mean movement (the time in intermediated activity); *mf* > *th* is counted as fast movement (the time in high activity). The sum of these counts is divided by the frames per second (*fps*) used to sample the video to obtain these values in time.

The user also sets a threshold for interaction (*ti*). The interaction is considered when the individuals approach a distance ≤ *ti*. The sum of all interactions of an individual is defined as centrality. The network density (*nd*) is a measurement associated with group interaction (Equation (15)). A network is a set of items in which the vertices are defined as nodes (*n*), and the connections among them are defined as edges (*m*) [34]. Here, the nodes are the individuals, and the edges represent the number of interactions among them.

(15) $$nd=\frac{2m}{n\left(n-1\right)}.$$

If the user defines a region of interest (*ri*), Ethoflow computes how long the individuals stayed inside this region, considering the position (coordinates *x*, *y*) of each individual in the video frames (*f*):

(16) $$\overset{f}{\underset{i=1}{\sum }}\left(x_{i},y_{i}\right)\in ri.$$

Considering the direction unit (*u*) of the individuals (*i*), the proportion of the group polarized (*p*) at each frame is calculated as

(17) $$p=\frac{1}{i}\left|\overset{i}{\underset{j=1}{\sum }}u_{j}\right|.$$

The angular momentum (rotate; *r*) for each frame is a cross product (or vector product; ×) between the distance (*d*) of an individual to the center of mass of the group and the direction of movement (*u*):

(18) $$r=\frac{1}{i}\left|\overset{i}{\underset{j=1}{\sum }}u_{j}\times d_{j}\right|.$$

These parameters provide information on the global structure of the group [35], such as how much individuals are aligned in a group (polarization*; gp*), how much the group displays low directional alignment between neighboring individuals (swarming*; gs*), and how much the group moves around its center of mass (milling*; gm*). The sum of these counts is divided by the *fps* to obtain these values in time:

(19) $$gp=\frac{\sum^{\;}p>0.65\;\mathrm{and}\;r<0.35}{fps},$$

(20) $$gs=\frac{\sum^{\;}p<0.35\;\mathrm{and}\;r<0.35}{fps},$$

(21) $$gm=\frac{\sum^{\;}p<0.35\;\mathrm{and}\;r>0.65}{fps},$$

#### 3.1.8. Complex Behavior Model

**Figure 3:**
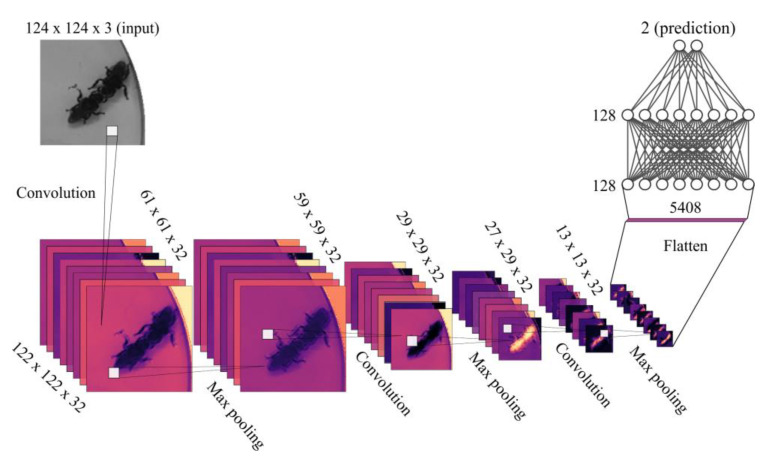
Convolutional neural network architecture defined after hyperparameter optimization (Appendix B) to recognize animal complex behavior on Ethoflow. This model was configured with stochastic gradient descent (learning rate at 0.0001 and momentum at 0.9) as an optimizer and binary cross-entropy as a loss function. Batch normalization was applied before max-pooling layers. Dropout was also applied after dense inner layers. In the inner layers (convolutional or dense), the function activation was Elu. The dimensions (width × height × depth) of feature map are given in each layer; the output dimensions of a layer are the same as the input dimensions of the next layer. In the flatten process, the data are transformed into a vector to enter the dense layers. In the last dense layer, a sigmoid function is applied, which gives the binary output.

Ethoflow also measures complex behaviors using a CNN model (step 1.8). Different hyperparameter configurations were tested to define the CNN model (Figure 3) (Appendix B). In this step, the bounding box computed from animal contours passes through the convolutional base (convolutional and max-pooling layers) for feature extraction. The activation function is applied to the output of each layer to introduce nonlinearity. Then, behavior classification is performed in the dense layers. When the complex behavior that the user is evaluating occurs, the network output will be equal to behavior 1; otherwise, behavior 0. The behavior occurrence sum is divided by the video frames to generate the percentage of occurrence of the behavior. Thus, we are interested in determining the occurrence of binary behaviors that are detectable through spatial information.

#### 3.1.9. Output

In step 1.8, the behavioral parameters are automatically saved in a comma-separated values (csv) file in the path defined by the user. This file also contains the raw data, the coordinates (*x, y*) of movement in each frame. Thus, the user is free to calculate other kinematic parameters, in addition to those automatically computed by the software. At the end of the video processing, Ethoflow exhibits the detection rate (*dr*), which is the proportion that the individual was detected during the entire video minus false detection. False detection is considered when an individual has between frames velocity greater than the percentile at 95% of group velocity across all frames. Given the instantaneous speed vector $IS=\left(is_{1},\ldots ,is_{n}\right)$ and f frames in the video, *dr* is defined as:

(22) $$dr=1-\left(\left(\frac{\sum_{i=1}^{f}is_{i}>2*P_{.95}\left(IS\right)}{is_{i}}\right)\left(\frac{1}{f}\right)\right),$$

where *P*._95_ is the percentile at 95% of the *IS* vector.

### 3.2. Applications and Performance

#### 3.2.1. Application in Heterogeneous Environments

**Figure 4:**
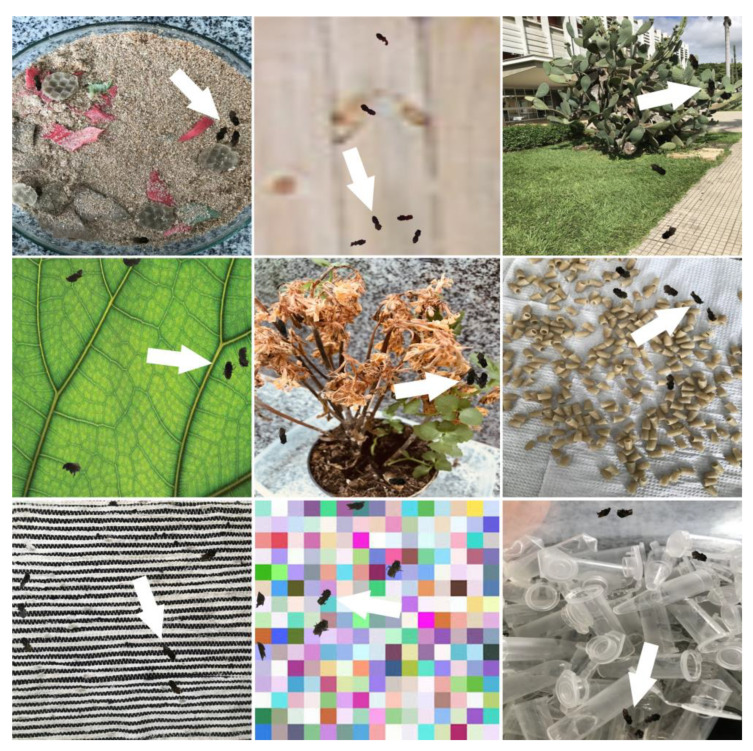
Examples of some images that were generated automatically to train the model for tracking the stingless bee *Melipona quadrifasciata* in different conditions of a heterogeneous background. The white arrows indicate some bee contours pasted in the backgrounds.

**Figure 5:**
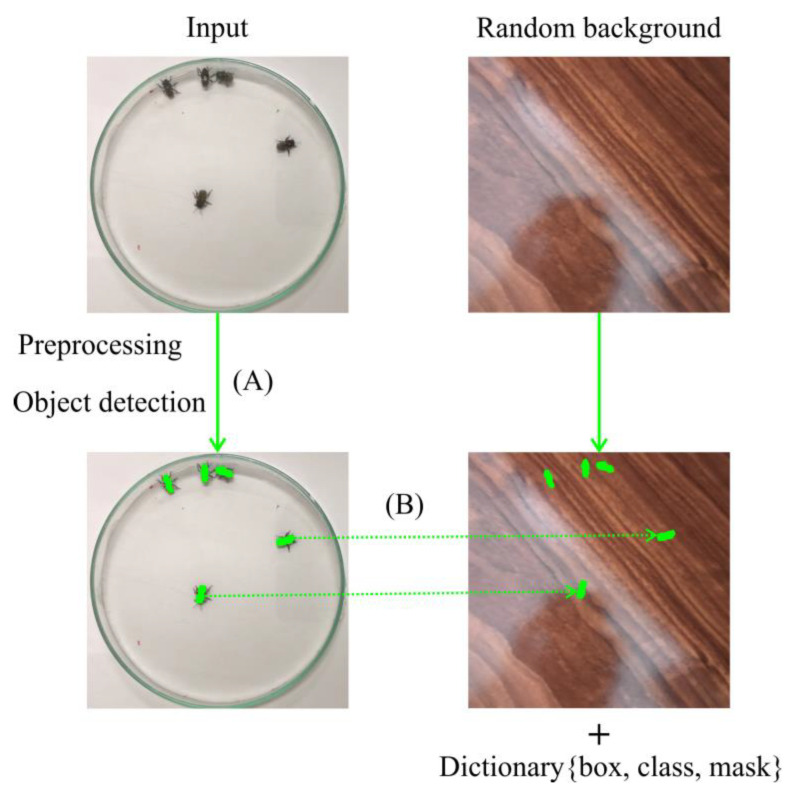
Schematic representation of the heuristic used to generate labeled images for the IS model automatically. The segmented objects (indicated by green masks) on a homogeneous background (**A**) are glued to random backgrounds (**B**).

The Ethoflow was run on a machine with Intel i7-9750H CPU 2.60 GHz × 12, 8 GB RAM and GPU NVIDIA^®^ GeForce^®^ GTX 1660 (6 GB) Ti Max-Q. To apply Ethoflow in a heterogeneous environment experiment, we trained the IS model to detect the bee *Melipona quadrifasciata* through the 1325 images in various heterogeneous backgrounds (Figure 4). In addition to these image data, the inputs with bounding box positions, classes, and masks (pixel-wise positions of the animals) are required to train the IS model [9]. The manual generation of these inputs is a laborious task. Then, we developed a heuristic to automatically generate these inputs based on several random backgrounds and a video in homogeneous conditions to detect objects using manual segmentation or Otsu’s method. Frames are randomly sampled in the video and pass through the algorithm’s preprocessing and object detection stages (Figure 5A). Then, the animals are “copied,” and the contours are “pasted” into random backgrounds (Figure 5B). Concomitantly, the bounding box, class, and mask of each animals are saved in a dictionary with the following structure: Dictionary {image_i_: {object_j_: {box: {center: {x,y}, width, height}; class:{target}; mask:{all points (x,y)}}}}.

Of all the data generated with the heuristic, 976 (74%) were used for training, 249 (19%) for validation, and 100 (7%) to evaluate the classification using the average precision (*AP*) [36]. To obtain *AP*, we calculated the intersection over union (*IoU*) of the predicted bounding boxes (i.e., the *x, y* coordinates in the upper-left corner and width and height of the rectangular box around the object of interest) and target bounding boxes. Based on the *IoU*, the precision (Equation (23)) and recall (Equation (24)) can be calculated using the true positives (*TP*), false positives (*FP*), and false negatives (*FN*) for the detected objects (*DO*) in a determined threshold (*x*) (Equation (25)).

(23) $$\mathrm{precision}=\frac{TP}{TP+FP}.$$

(24) $$\mathrm{recall}=\frac{TP}{TP+FN}.$$

(25) $$\left\{\begin{array}{c} \mathrm{if}\;IoU\;\geq \;x,\;DO=TP\\ \mathrm{if}\;IoU\;<\;x,\;DO=FP\\ \mathrm{if}\;\mathrm{the}\;\mathrm{model}\;\mathrm{fails}\;\mathrm{to}\;\mathrm{detect}\;a\;\mathrm{target}\;\mathrm{object},\;DO=FN \end{array}\right\}$$

There is a tradeoff between the precision and recall, wherein the higher the recall, the more the model tends to find all the target objects, i.e., a low *FN* value. However, an increase in the recall tends to decrease the precision, as it increases *FP*. Considering equally spaced recall levels n=(0, 0.1,⋯, 1.0), interpolation is performed using the highest precision value for a given recall. Then, the *AP* is obtained from the interpolated values of the precision $\left(P_{interp}\left(r\right)\right)$:

(26) $$AP=\frac{1}{n}\overset{n}{\underset{i=0}{\sum }}P_{interp}\left(r_{i}\right).$$

#### 3.2.2. Application in Complex Behavior

**Figure 6:**
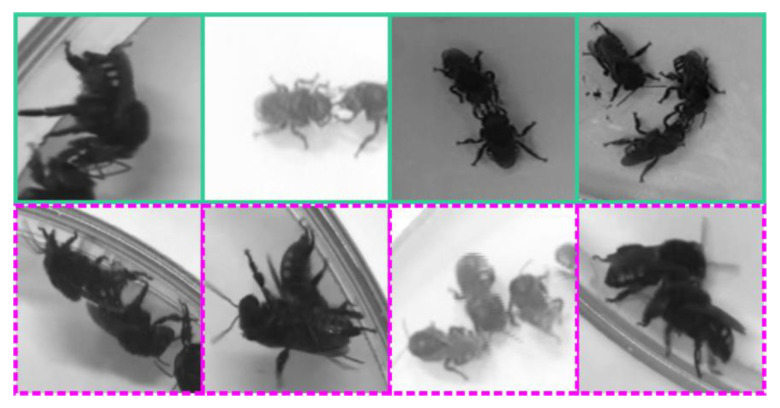
Examples of images that were automatically labeled with our heuristic to train the CNN model to recognize trophallaxis in the stingless bee *Melipona quadrifasciata*. The images with green outlines (**top**) are examples of trophallaxis. The images with dashed purple outlines (**bottom**) are examples of non-trophallaxis.

Ethoflow was also applied to learn the detection of trophallaxis, the complex social behavior of food exchange among nestmates, in *M. quadrifasciata.* Thus, 1270 labeled images were generated (724 for non-trophallaxis and 546 for trophallaxis) (Figure 6). In this dataset, 70% of the data was used for training, while 20% was used for validation. Another sample dataset (10%) was used to assess the classifier’s performance based on the global accuracy from the confusion matrix, Kappa index, and Z-test (5%).

The labeled images used to train the CNN model for recognizing trophallaxis were also generated through a heuristic automatically. When bees perform trophallaxis, they position themselves in front of each other and exchange food. Based on this predictable positioning, the heuristic was based on the individuals’ area and body length. Initially, the program estimates the median (*M*) and standard deviation (*sd*) of the body area (*a*) and length (*l*) in frames where there is no crossing (no meeting between individuals). Subsequently, the software obtains the images (*b*) from the video and labels them as trophallaxis *if*:

(27) $$\left\{\begin{array}{c} area\left(b\right)⩾2*\left(M\left(a\right)-sd\left(a\right)\right)\;\mathrm{and}\\ area\left(b\right)⩽2*\left(M\left(a\right)+sd\left(a\right)\right)\;\mathrm{and}\\ length\left(b\right)⩾2*\left(M\left(l\right)-sd\left(l\right)\right)\;\mathrm{and}\\ length\left(b\right)⩽2*\left(M\left(l\right)+sd\left(l\right)\right) \end{array}\right\},Else:b\;\mathrm{is}\;\mathrm{not}\;\mathrm{trophallaxis}.$$

#### 3.2.3. Application in Behavioral Bioassays

A behavioral assay was performed with the two stingless bee species. Bees of both species were collected from four colonies each of *M. quadrifasciata* and *Partamona helleri* in Viçosa, State of Minas Gerais, Brazil (20°45′ S and 42°52′ W). The collected bees were kept for 1 h in the laboratory under conditions similar to those found in their colonies (28 °C and 80% relative humidity in total darkness) [37]. Subsequently, bee behavior was recorded in the arenas (Petri dish, 9 cm diameter, 2 cm height) for 15 min with a digital video camera (HDR-XR520V, Sony Corporation) at 30 fps and high definition (1920 × 1080 pixels). Behavioral bioassays were performed in a room with artificial fluorescent light at 25 ± 3 °C and 70 ± 5% relative humidity. Bioassays were performed with 37 replicates, with each replicate corresponding to a group of five bees of each of the two species. The kinematic variables measured with Ethoflow included centrality, polarization, milling, resting, meandering, and tracked distance. In the centrality response, the interaction was considered when the individuals approached a distance ≤1.41 cm. An instantaneous tracked distance ≤0.046 cm frame^−1^ was counted as resting. Centrality was the response variable in the model with interaction between polarization and bee species, or model with interaction between milling and bee species. Meandering was the response variable in the model with interaction between resting and bee species. Besides, the tracked distance between the bee species was compared. These models were fitted with generalized linear models (GLM) with a gamma distribution, displaying adequate distribution for continuous data in which the variance increases with the square of the mean [38]. When an explanatory variable had no significant effect, the model was simplified, and the results were plotted as a function of the significant variable.

A toxicological bioassay was also performed with *M. quadrifasciata* to demonstrate trophallaxis recognition under pesticide stress conditions. The acclimated bees were orally exposed to the commercial formulation (cf) (water-dispersible granules at 700 g a.i. Kg^−1^, Bayer CropScience, São Paulo, SP, Brazil) of the neonicotinoid imidacloprid in a sublethal concentration (0.2 mg cf L^−1^). This concentration is 300× smaller than that recommended for controlling the whitefly *Bemisia tabaci* (60 mg cf L^−1^) [39]. The pesticide imidacloprid is commonly associated with bee decline and causes motor impairments in bees [40]. After 3 h of exposure, the bees were filmed as previously described, and trophallaxis behavior was quantified using Ethoflow. Trophallaxis response (*n* = 60) to the pesticide was assessed using a GLM with a Poisson distribution, a suitable distribution for count data [38].

#### 3.2.4. Performance

Using videos with variations in resolution, the number of individuals, animals, and backgrounds (Supplementary Materials; Figure S1), we evaluated some parameters associated with Ethoflow’s performance and also compared it with other tracking software that has a satisfactory processing rate, based on the processing speed obtained by Sridhar et al. (2019) [13]. A multiple regression model was applied to assess whether the fps rate responds to the interaction between the resolution and the number of individuals. The effect of centrality and the number of individuals in fps was assessed using a GLM with a gamma distribution. Analysis of covariance (ANCOVA) was performed to assess whether the detection rate varied with the interaction between the number of individuals and background (homogeneous and heterogeneous).

## 4. Results

### 4.1. Heterogeneous Environment and Complex Behavior

**Figure 7:**
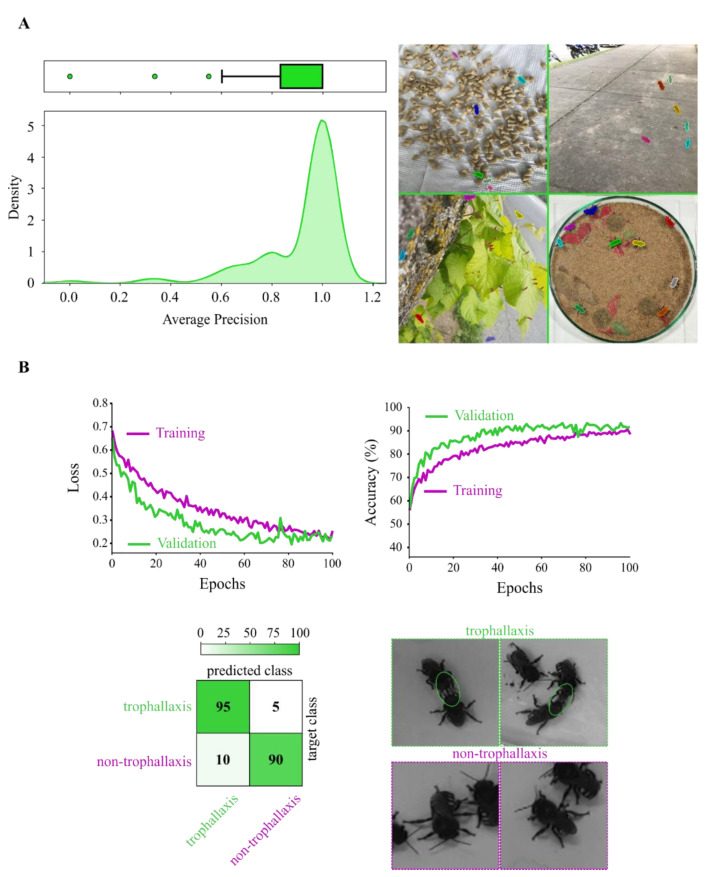
Performance of the AI models used in Ethoflow. (**A**) Animals’ detection in heterogeneous environments based on instance segmentation (IS). The high average precision (left panel; *n* = 100) implies that the model precisely detects real animals in the scenes with no false positives, as demonstrated by (right panel) the detected animals (marked *Melipona quadrifasciata* bees with masks in random colors) in different heterogeneous backgrounds. (**B**) The training process of the CNN model (top panel) and validation (percentage confusion matrix; bottom left panel) (*n* = 127) for the monitoring of trophallaxis (green circles) in bees (bottom right panel).

Ethoflow was efficient in detecting the tested bees with high precision and low false positives in heterogeneous environments (average precision ± standard error = 0.916 ± 0.02; Figure 7A). In addition, in complex behavior assessment, the CNN model exhibited high accuracy in the validation process (global accuracy = 92.13%, Kappa index = 0.84, Z = 24.74, Figure 7B).

### 4.2. Behavioral Bioassays

**Figure 8:**
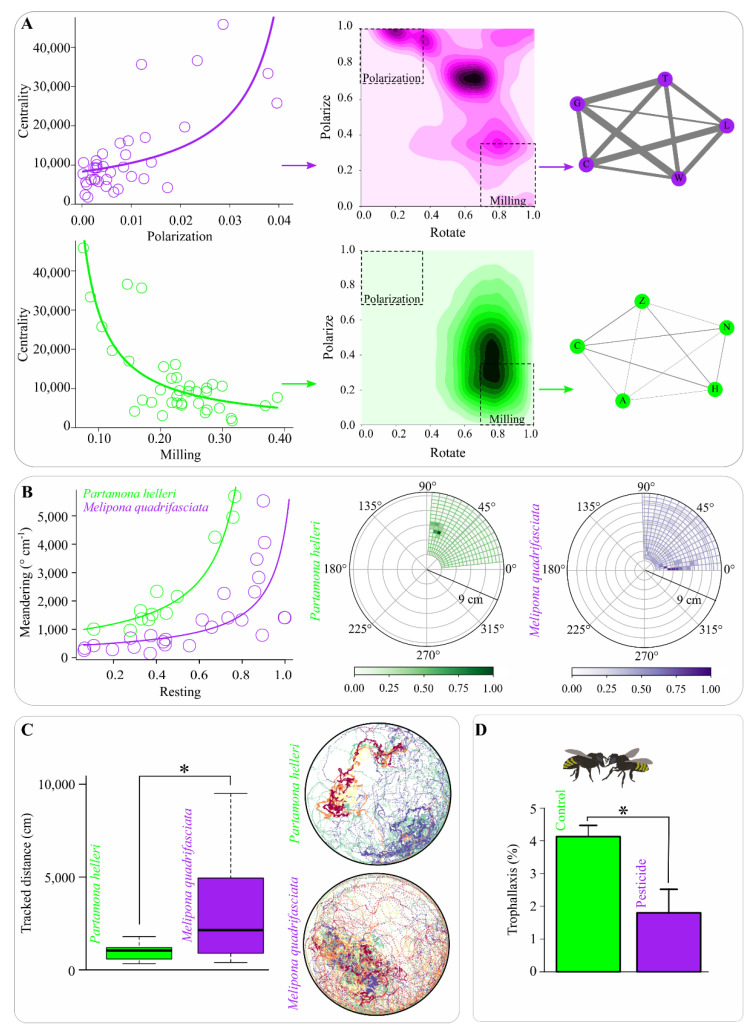
Behavioral assessments conducted using the Ethoflow software. (**A**) Association between centrality and group dynamics polarization (top panel) and milling (bottom panel) (*n* = 37). The 2D density plots and network diagrams showed that a higher interaction exists among individuals in more polarized bee groups, while this interaction is reduced in the milling groups. In the networks, the circles represent individuals, and connections correspond to the edges, where their widths are proportional to the frequency of interactions. (**B**) Meandering behavior is associated with resting proportions (left panel) (*n* = 37) and histograms of polar coordinates (rays and azimuth angles) for the two bee species (right panel). (**C**) The tracked distance of the assessed bee species (*n* = 37). In group representative tracks, the track color reflects the individual identity (right panel). (**D**) Trophallaxis alteration in *Melipona quadrifasciata* after pesticide exposure (*n* = 60). * *p* < 0.05 in the generalized linear model.

The results of the bioassays demonstrated significant differences between behaviors, bee species, and response to pesticide stress. In both species, the centrality increased with the polarization of the group (F _1, 35_ = 25.1, *p* < 0.0001) and decreased with milling (F _1, 35_ = 46.2, *p* < 0.0001) (Figure 8A). Meandering was influenced by the statistical interaction between the variables resting and bee species (F _1, 33_ = 4.71, *p* = 0.037; Figure 8B). Moreover, a difference between species was observed in the tracked distance (F _1, 35_ = 13.6, *p* = 0.0008; Figure 8C), and bees exposed to the pesticide exhibited significantly reduced trophallaxis (χ^2^ = 94.9, df = 58, *p* < 0.0001; Figure 8D).

### 4.3. Performance

**Figure 9:**
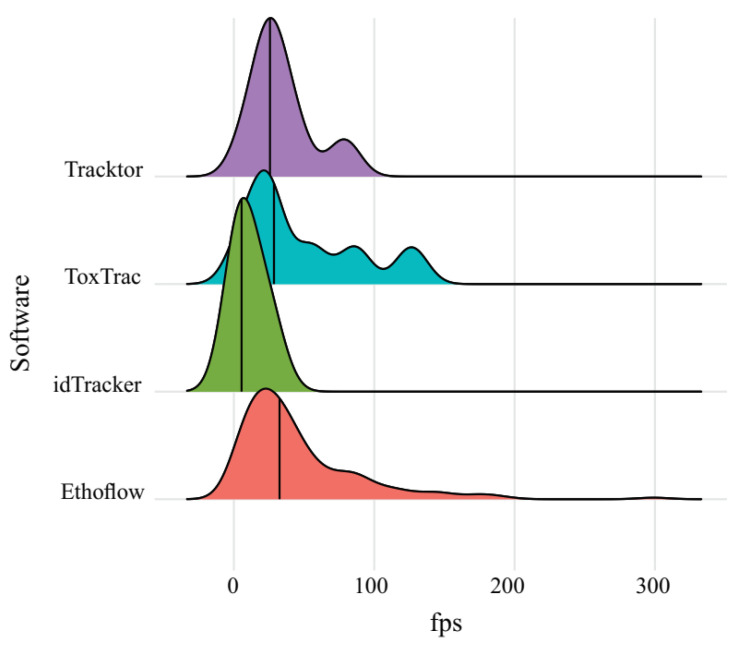
Distribution of frames per second rate exhibited for tracking software. The vertical lines within the density curves show the median.

In homogeneous backgrounds, Ethoflow achieved a median rate of 32.5 fps. This rate is a satisfactory processing speed compared to other tracking software that does not use AI in their algorithms (e.g., idTracker = 5.5 fps; ToxTrac = 28.6 fps; Tracktor = 25.7 fps) (Figure 9).

**Figure 10:**
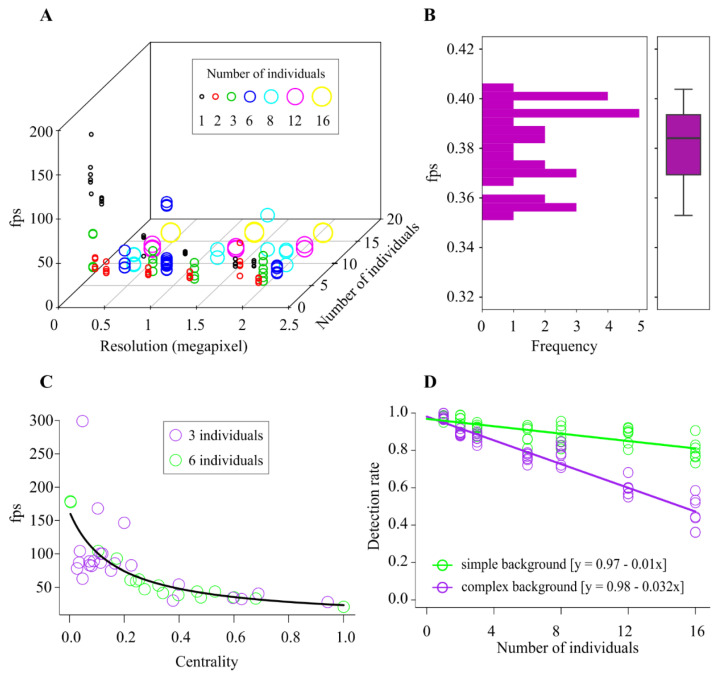
Quantification of the performance of Ethoflow. (**A**) Frames per seconds (fps) response to the video resolution (in pixels) and the number of individuals in homogeneous backgrounds; the dots (*n* = 134) represent the raw data. (**B**) Histogram of the fps in heterogeneous environments (*n* = 30). The box plot indicates the median and range of dispersion (lower and upper quartiles and outliers). (**C**) Fps in response to centrality. The proportion of group interaction per frame was used to quantify the centrality (*n* = 40). (**D**) Accuracy of the software as a function of the interaction between the number of individuals and type of environment (homogeneous and heterogeneous); the symbols represent the raw data (circles; *n* = 98).

Statistical interaction was observed between the variables video resolution and group size in fps rate (F _1, 130_ = 12.81, *p* = 0.0005, Figure 10A). The heterogeneous environment quantification was not influenced by the video resolution or number of individuals (F _1, 28_ = 0.81, *p* = 0.37, Figure 10B), and the fps rate in a heterogeneous environment (0.386) was lower than in homogeneous backgrounds. The fps decreased with an increase in the centrality of individuals (F _1, 38_ = 81.24, *p* < 0.0001, Figure 10C). There was no significant effect on the number of individuals (F _1, 37_ = 0.009, *p* = 0.93), and no interaction was observed between the centrality and individuals (F _1, 36_ = 1.62, *p* = 0.21). Besides, the software exhibited high detection rates with significant interaction between the number of individuals and type of background (F _1, 94_ = 137.85, *p* < 0.0001, Figure 10D), where an increase in the number of individuals had a greater influence on the heterogeneous environments.

## 5. Discussion

We developed Ethoflow software using computer vision, machine learning and deep learning techniques. This program had consistent speed rates and accuracy on processing. In addition to the possibility to study complex behaviors, Ethoflow allows multivariate assessment of kinematic behaviors. Multivariate assessment of behavioral traits can bring important insights into animals’ ecological aspects, for instance, in studies of toxicological assessments and animal behavior [41,42,43]. Some modern software programs that use deep learning to evaluate behaviors demand powerful machines with GPU [17,18,44], which makes the analysis of laboratory routines in ordinary computers difficult. In the Ethoflow algorithm, the AI processing is separate from the other modules, enabling kinematic measurements on an ordinary computer and assessing more complex behavior using a GPU. Wherefore, to perform kinematic measurements in homogeneous environments, an ordinary computer is sufficient (e.g., a central process unit of the 3.60 GHz) to run Ethoflow. In complex behavior assessments and heterogeneous environments, a GPU computer is interesting for optimizing speed-up computational processes.

Unraveling complex behaviors can be limited by software without adequate tools or software that are complex to set up or does not have a GUI, requiring familiarity with their tools [13,17,45], limiting their usage in the general public. Thus, there is a demand in research for powerful software with simplified-interface that, at the same time, increase the ability to study more complex behaviors. In this context, the Ethoflow software looks friendly and does not require line commands to be used due to the GUI. Additionally, Ethoflow does not require a great familiarity with computational tools and has multidisciplinary applications.

During the processing in homogeneous backgrounds, the effects of resolution and number of individuals in the fps demonstrated that the frame reading step (higher resolution, higher reading time) and calculating identities (more individuals, more combinations) could decrease the processing speed. Nonetheless, the implementation of multi-threaded reading [46] in Ethoflow solves these problems. This type of reading avoids the delay between calculating the identity and reading the frames, whereby there will always be frames available in the queue for immediate calculation of the identity. This procedure possibility satisfactory fps rates compared to other software available for the same purpose [13,14,15]. The identity calculation algorithm step occurs when at least two individuals interact. Thus, fps showed a negative correlation with centrality because the greater the interaction, the greater the identity calculations. The number of individuals (mainly in groups > 3) had no binding effect on the fps, probably, because the amount of interaction between individuals depends on the density of the group (i.e., number of individuals per space) and not only on the size (i.e., the number of individuals) [35].

In heterogeneous environments, there is no influence of the video resolution or number of individuals on the processing, and the fps rate is lower than in homogeneous backgrounds. This shows that the main bottleneck in processing occurs in the detection of animals by Ethoflow through the instance segmentation model. With instance segmentation, real-time processing (~30 fps) has not been achieved; processing around 5 fps was reported using a robust GPU [9]. Even though it is not possible to achieve real-time processing with instance segmentation, this functionality in the Ethoflow imposes great advantages given the various possibilities of analysis in heterogeneous environments. Furthermore, video acquisition by Ethoflow is independent of processing, which enables real-time video records.

The reliable detection rates obtained with Ethoflow demonstrated that this software is sufficiently robust for applications in different assays. Moreover, using the heuristic to generate training data automatically made it possible to obtain a high average precision model. Such in heterogeneous environments, there was a more pronounced decrease in the detection rate of objects; therefore, increasing the amount of data for training can improve the detection [47]. With the use of our heuristic, increasing the amount of data does not take much time from the user, but it could increase the time of computational training and inference. Another alternative would be to increase the quality of the data with images annotated manually. One tool that can be used to label images manually is VGG Image Annotator (VIA) [48].

## 6. Conclusions

This study provides information about the development of e-applications of computer vision and the artificial intelligence-based software Ethoflow. This software is suitable for multivariate kinematic evaluations, behavioral assessments in heterogeneous environments, tracking individuals in groups maintaining their identities, and can be trained to learn complex behaviors. Ethoflow was applied to biological assessments and was efficient to detect significant differences between different bee species and pesticide stress. Some possibilities of data analysis and representation were demonstrated with Ethoflow’s output. The deep learning models were implemented to expand the possibilities of animal behavior analyses to other fields, including the behavioral monitoring of domestic animals in precision livestock farming. According to demand, Ethoflow will be constantly updated for future improvements and new functions, such as tracking three dimensions. Therefore, Ethoflow is a helpful support tool for technical and scientific applications in biology and related fields.

## 7. Patents

This software is registered with the Brazilian National Institute of Intellectual Property (INPI, Ministério da Economia, Brazil, reg. no. BR 51 2020 000737-6).

## Figures and Tables

**Figure A1:**
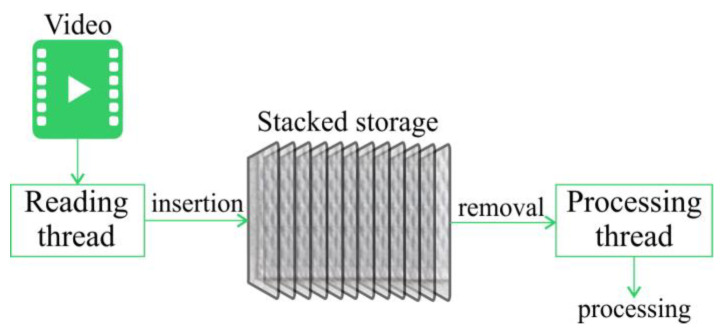
Representation of the processing steps in the multi-threading feature of Ethoflow.

**Figure A2:**
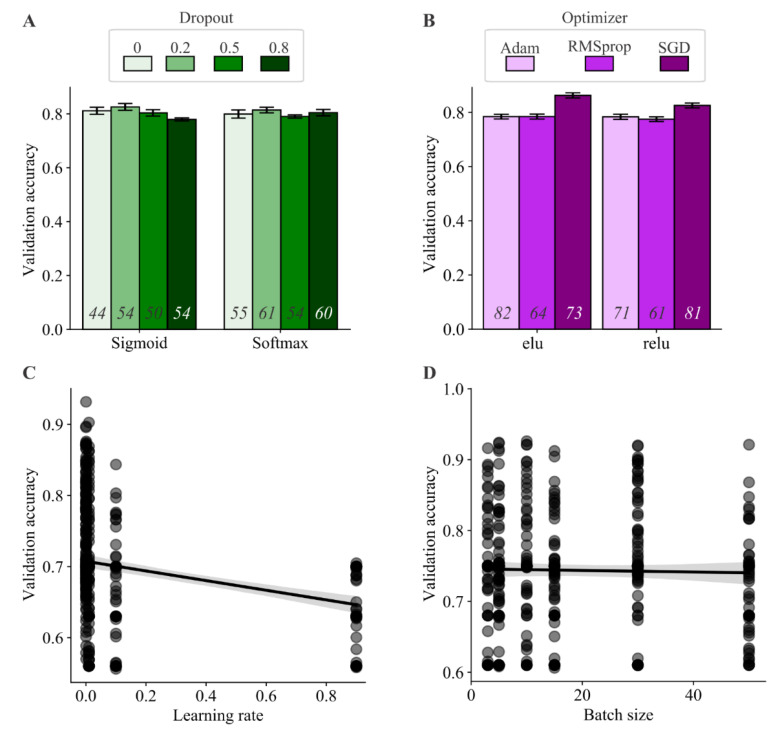
Hyperparameter optimization of the CNN model used in Ethoflow. (**A**) Compilation of the validation accuracy in response to the dropout and the activation function of the network output. (**B**) Validation accuracy in response to the optimizer and activation function of the inner layers. (**A**,**B**) The bars represent the mean ± standard error. The values at the base of each bar represent the number of times a given configuration was tested. Scatterplot of the accuracy as a function of the (**C**) learning rate and (**D**) batch size. The translucent band around the line of regression represents the confidence interval (*n* = 432).

## Data Availability

Ethoflow-related files, including source code and video tutorials, are available at https://sites.google.com/view/ethoflow (accessed on 6 May 2021). The datasets generated for this study are available at https://github.com/bernardesrodrigoc/Ethoflow (accessed on 6 May 2021).

## Supplementary Materials

The following are available online at https://www.mdpi.com/article/10.3390/s21093237/s1, Figure S1 and Table S1.

## Appendix A

The video is read by a thread that is independent of the processing thread, and the frames are stored in a stack (queue). This queue is a linear data structure that stores items in a “FIFO” (First In, First Out) manner (Figure A1). Frames are exchanged between the reading and processing threads. This increases the processing speed, as frames are always present in the queue and ready for processing, and no time is spent waiting for the next frame to be read.

## Appendix B

Different hyperparameters were tested to find a suitable convolutional neural network (CNN) model. This model was trained to recognize trophallaxis in the stingless bees, *Melipona quadrifasciata*, and can be used to recognize many binary behaviors. Using the validation accuracy of such a variable response, the interaction between the dropout and the activation function of the network output was evaluated (Figure A2A). Dropout is a regularization technique that randomly zeros out the input units of a layer, breaking fixed patterns to avoid overfitting [49]. Here, a better response was obtained, deactivating neurons with a probability of 0.2 (dropout = 0.2) (Figure A2A). The higher rates, despite reducing overfitting, decreased the accuracy. The activation function of the network output that presented the best response was the sigmoid function (Equation (A1)) (Figure A2A). This function binarizes the network output (0 or 1). As it involves a binary behavior classification, the sigmoid function is expected to generate better output.

(A1) $$\left\{f\left(x\right)=\frac{1}{1+e^{-x}},\;\mathrm{where}\;x\;\mathrm{is}\;\mathrm{the}\;\mathrm{output}\;\mathrm{from}\;\mathrm{the}\;\mathrm{previously}\;\mathrm{hidden}\;\mathrm{layer}\right\}.$$

The activation function of the inner layers and the optimization method were also evaluated. The best result was obtained with the exponential linear unit (Elu) function, along with mini-batch stochastic gradient descent (mini-batch SGD) as the optimizer (Figure A2B). The Elu function (Equation (A2)) is an identity function for positive values, and it tends smoothly to −α for negative values. This function saturates for very small (extremely negative) values, resulting in the activation average being close to zero. Thus, ELUs tend to normalize the layer’s output, accelerate learning, and increase accuracy [50].

(A2) $$f\left(x\right)=\left(\begin{array}{c} x,\;if\;x>0\\ \alpha *(e^{x}-1),\;if\;x⩽0 \end{array}\right).$$

In the mini-batch SGD, the term stochastic refers to a random sampling of batches in the data. Based on the loss value, the optimizer plays the role of updating the network’s trainable parameters (weights). This is executed by calculating the loss gradient concerning the parameters (current weights) of the network. Mathematically, this process is performed by deriving the cost function and finding the gradient of the current weights. Then, the weights are updated in the gradient’s opposite direction, reducing the loss slightly with each batch. Since the classification is binary (the output from the network is a probability), binary cross-entropy (Equation (A3)) was used as the cost function. Cross-entropy is a measure of the distance between the expected result *y* and the predictions p(y).

(A3) $$H_{p}\left(q\right)=\frac{-1}{n}\overset{n}{\underset{i=1}{\sum }}y_{i}*log\left(p\left(y_{i}\right)\right)+\left(1-y_{i}\right)*log\left(1-p\left(y_{i}\right)\right),$$

where *n* is the number of network outputs.

As an increase in the learning rate tended to decrease the accuracy (Figure A2C), the lowest rate tested (0.0005) was maintained. The learning rate determines the magnitude of gradient descent. At high learning rates, network updates can result in great randomness.

The network interacts with the data in mini-batches, i.e., it does not process an entire dataset simultaneously; rather, the data is divided into small batches. Although this hyperparameter is important in CNN models [51], it does not play an important role in our model (Figure A2D). Therefore, one of the smallest values (batch size = 5) was selected to accelerate the network’s training time.

In many statistical models, the normalization of variables is important (e.g., to avoid the predominance of some variables due to different scales). To this end, batch normalization layers were used in the CNN model. This layer can adaptively normalize the data as the mean and variance change during training [52].

Using the hyperparameters defined above, the network’s size (number of layers) was also evaluated, and better accuracy was obtained with smaller architectures (Table A1). While more layers (a higher-dimensional representation space) allow the network to learn more complex representations, this increases the network’s computational cost; accordingly, model L7 was employed.

**Table A1:** Different architectures tested to ascertain the ideal number of layers in the CNN model (*n* = 28).

	Number of Layers		
Validation Accuracy (Mean ± sd)	Convolutional	Dense	Model
0.63 ± 0.028	5	4	L1
78 ± 0.036	5	3	L2
0.83 ± 0.042	5	2	L3
0.81 ± 0.063	4	4	L4
0.8 ± 0.121	4	2	L5
0.8 ± 0.020	3	4	L6
0.91 ± 0.031	3	3	L7

Data augmentation is a powerful technique for mitigating overfitting. Using the defined architecture (model L7 in Table A1), different data augmentation configurations were tested (Table A2). Excessive data augmentation reduces the accuracy, while sets with little augmentation increase overfitting. Thus, set 3 was deemed the best option to address the problem of overfitting.

**Table A2:** Sets tested for data augmentation. In all the tests, the horizontal flip and fill mode = the “nearest” was used. Model L7 in Table A1 was used for these tests.

Parameters	Set 1	Set 2	Set 3	Set 4
Rotation range	20	16	14	11
Width shift range	0.1	0.08	0.06	0.01
Height shift range	0.1	0.08	0.06	0.01
Shear range	0.05	0.02	0.01	0.008
Zoom range	0.1	0.08	0.06	0.01
