# Supplementary material for: Ethoflow: Computer Vision and Artificial Intelligence-Based Software for Automatic Behavior Analysis
Source: Sensors (Basel). 2021 May 7;21(9):3237. doi: 10.3390/s21093237 (PMC8124799; doi:10.3390/s21093237)
Supplement: Supplementary file 1 [file sensors-21-03237-s001.zip › sensors-1194717-supplementary.pdf]

Supplementary Materials

Ethoflow: Computer Vision and Artificial Intelligence-Based Software for Automatic Behavior Analysis

Rodrigo Cupertino Bernardes [1,*], Maria Augusta Pereira Lima [2], Raul Narciso Carvalho Guedes [3], Clíssia Barboza da Silva [4] and Gustavo Ferreira Martins [5]

1 Department of Entomology, Federal University of Viçosa, Viçosa, MG 36570-900, Brazil; bernardesrodrigoc@gmail.com
2 Department of Animal Biology, Federal University of Viçosa, Viçosa, MG 36570-900, Brazil; maugusta@ufv.br
3 Department of Entomology, Federal University of Viçosa, Viçosa, MG 36570-900, Brazil; guedes@ufv.br
4 Laboratory of Radiobiology and Environment, University of São Paulo-Center for Nuclear Energy in Agriculture, 303 Centenário Avenue, Piracicaba SP 13416-000, Brazil; clissia_usp@hotmail.com
5 Department of General Biology, Federal University of Viçosa, Viçosa, MG 36570-900, Brazil; gmartins@ufv.br
* Correspondence: bernardesrodrigoc@gmail.com; Tel.: +5531995169902

Table S1. Summary of the main input and output files, formats, description, and quick examples of how to use these files.

	**File**	**Format**	**Description**
**Input**	Video	mp4, avi, mov, mpeg, flv, wmv	A video to be analyzed
	CNN model	h5	If analyzing complex behavior, it is necessary to load a trained model (usually a keras model). As a default, Ethoflow goes with a trained model to detect trophallaxis on the stingless bee *Melipona quadrifasciata* (our model animal).
	Random backgrounds	bmp, jpeg, jpg, png, pbm, ras, tiff, tif, hdr, pic	If using the heuristic to generate labeled images for the instance segmentation, it is necessary to load various random backgrounds. As a default, in the path "Ethoflow/make_mask_rcnn_data/backgrounds", there are various random background images.
**Output**	Result data	csv	A file containing all variables computed by Ethoflow. This file also contains the raw data, which are the coordinates (*x*, *y*) of movement in each frame. In the Ethoflow webpage, we provided a Jupyter notebook with an example of how to read and plot the tracking of individuals.<br>Visit: https://sites.google.com/view/ethoflow/scripts
	Video	mp4	Processed video showing the identity trail of each individual in random colors
	Raw data	gml	A file containing information about the structure of the interaction network among animals. In the Ethoflow webpage, we provided a Jupyter notebook with an example of how to read and analyze this file.<br>Visit: https://sites.google.com/view/ethoflow/scripts

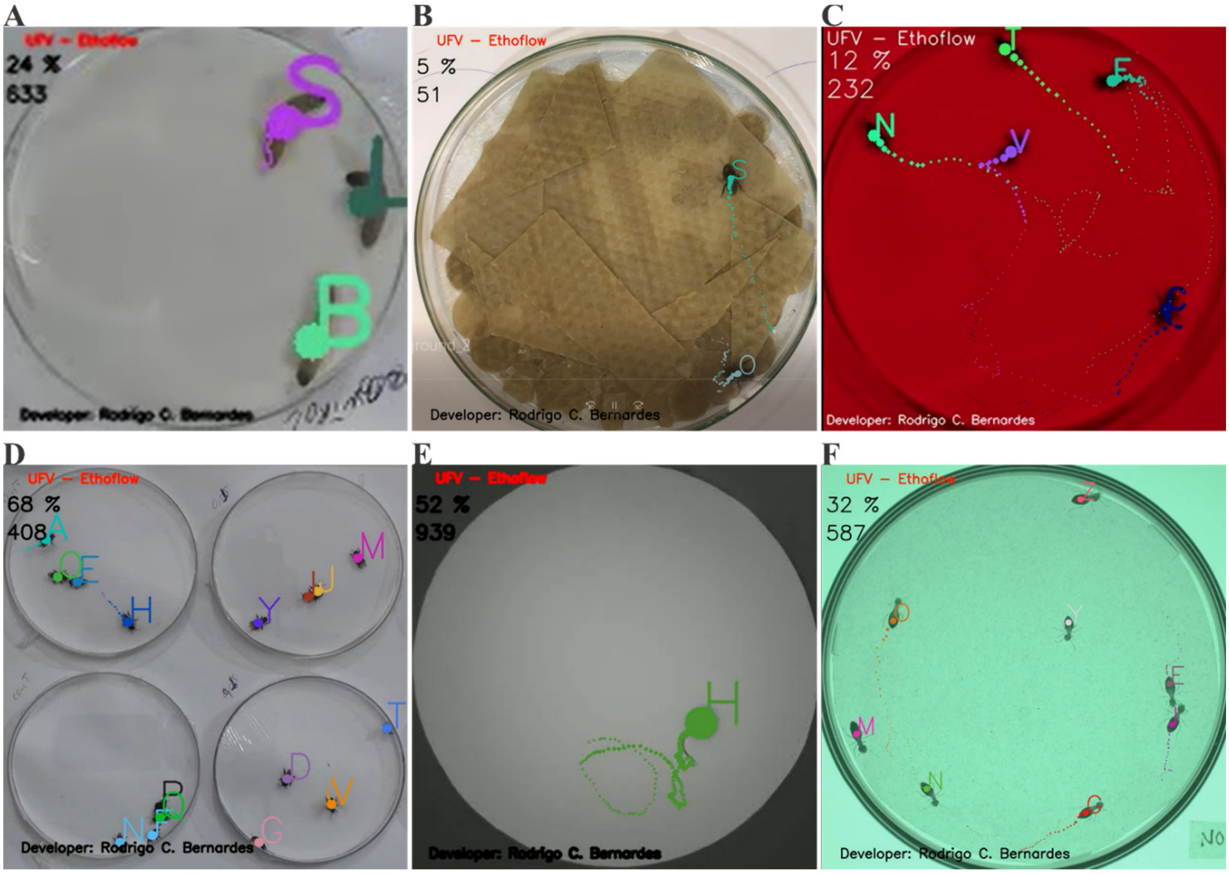

Figure S1. Examples of videos processed with Ethoflow. (A) Monitoring of *Galleria mellonella* larvae in the low-quality video. (B), (C) and (D) Monitoring bees (*Melipona quadrifasciata* and *Partamona helleri*) in different background and luminosity conditions. Monitoring of the C57Bl/6 mouse strain (E) and termites (*Constrictotermes cyphergaster*) (F). (E) and (F) were obtained from the freely available GitHub repository (https://github.com/vivekhsridhar/tracktor/tree/master/examples). Some videos processed with Ethoflow are available at https://github.com/bernardesrodrigoc/Ethoflow/tree/master/videos_example_ethoflow; DOI: https://doi.org/10.5281/zenodo.3956831.
